# Gender differences in gaze patterns to Instagram influencer body parts: predicted by subjective body image perception and negative affect

**DOI:** 10.3389/fpsyg.2026.1857912

**Published:** 2026-07-02

**Authors:** Kyulee Shin, Youngirl Jeon

**Affiliations:** 1 Department of Sports Sciences, Seoul National University of Science & Technology, Seoul, Republic of Korea; 2 Research Institute for Physical Fitness and Sport Science, Sungkyunkwan University, Suwon-si, Republic of Korea

**Keywords:** body image perception, eye tracking, gaze patterns, gender difference, influencer, Instagram, negative affect

## Abstract

**Introduction:**

This study examined gender differences in visual attention to Instagram sports and health influencer body images and explored how subjective body image perception (BIP) and negative affect predict gaze patterns.

**Methods:**

Sixty healthy Korean adults (30 males, 30 females) with a normal-range BMI (18.5–22.9 kg/m²) viewed 15 photographs of same-sex Korean social media influencers for 20 seconds each (300 seconds total) while eye movements were recorded using a Tobii Pro Fusion eye tracker (120 Hz). Nine body areas of interest (AOIs) were defined: face, chest, arm, abdomen, hip, thigh, shin, calf, and trunk back.

**Results:**

A significant Gender × AOI interaction was found for both fixation duration and fixation frequency (both *p* < .001). Males showed greater fixation on the chest and arm, whereas females fixated more on the face and hip. BIP variables showed significant gender main effects but no significant change following exposure. Negative affect (PANAS-N) decreased significantly after influencer exposure (*p* < .05), particularly for upset, irritable, and distressed items. After false discovery rate (FDR) correction, BIP discrepancy was negatively correlated with chest fixation in males (*r* = −.563), whereas pre-exposure upset (PANAS2) positively predicted fixation on the chest and trunk back in females (*r* = .524–.535). Regression analyses confirmed these gender-specific pathways.

**Discussion and Conclusion:**

These exploratory findings suggest that gaze patterns toward influencer body parts differ by gender, with body image cognition more prominent in males and affective state more prominent in females. Future confirmatory research incorporating direct measures of body surveillance, social media use, and muscularity drive is needed to validate these patterns.

## 1 Introduction

The rapid growth of social media has exerted wide-ranging effects on psychological processes related to body image (Castellanos Silva and Steins, 2023; Czubaj et al., 2025; Sanzari et al., 2023; Seekis et al., 2020; Thai et al., 2024). Instagram, with over 3 billion users worldwide, has become a highly visual platform and a major media environment in which idealized body images are routinely consumed (Casale et al., 2024; Jánská et al., 2024; Pedalino and Camerini, 2022). Sports and health influencers active on Instagram continuously share their physique and lifestyle, potentially exerting substantial influence on the body image and psychological well-being of their followers. Unlike established celebrities or advertising models, influencers are content creators with whom users form parasocial relationships characterized by psychological closeness, and they may function as a distinctive reference group that normalizes idealized body standards (Perloff, 2014). Eye-tracking research has demonstrated that Instagram content can shape viewers’ visual attention in ways that reflect underlying body image concerns and social comparison tendencies (Jabłońska, 2024; Lindholm et al., 2021).

The relationship between media and body image is supported by extensive meta-analytic evidence. A meta-analysis by Grabe et al. (2008), synthesizing 77 studies and 141 effect sizes, found that exposure to media images depicting the thin ideal was significantly associated with body dissatisfaction (ds = −0.28), thin-ideal internalization (ds = −0.39), and eating-related attitudes (ds = −0.30) in women. Holland and Tiggemann (2016) synthesized 20 studies and concluded that SOCIAL MEDIA use was significantly associated with body image concerns and disordered eating, with photo browsing, posting, and appearance-related feedback seeking being especially strongly linked to body dissatisfaction. These meta-analytic findings indicate that media exposure effects are small to moderate in magnitude (Grabe et al., 2008), while highlighting that individual psychological characteristics play an important moderating role. Recent meta-analyses and systematic reviews have consistently reported that SOCIAL MEDIA use frequency, appearance comparison tendency, and body image internalization are closely associated with body dissatisfaction (Harriger et al., 2023; Xu et al., 2025).

However, the evidence is not uniformly consistent. Several studies have found null or even positive effects of social media body image exposure under specific conditions. For example, exposure to body-diverse or body-positive content has been associated with improved body satisfaction (Stein et al., 2024; Ogden et al., 2020), and the perceived attainability of comparison targets moderates whether upward social comparison produces negative or positive affective outcomes (Festinger, 1954). Harriger et al. (2023) caution that effect sizes across studies are small to moderate and are likely moderated by individual difference variables, platform-specific features, and content type. These inconsistencies underscore the need for research that moves beyond general social media use toward examining specific content types (e.g., fitspiration vs. body-positive), specific psychological mechanisms (e.g., cognitive vs. affective), and objective behavioral measures such as eye tracking, rather than relying solely on self-report.

One particularly prominent content type in the SOCIAL MEDIA environment is the fitspiration image produced by sports and health influencers. Fitspiration refers to an online content trend aimed at inspiring healthier lifestyles through the promotion of exercise and healthy behaviors (Bowles et al., 2021; Mayoh and Jones, 2021; Raggatt et al., 2018). Although fitspiration content ostensibly promotes a healthy aesthetic distinct from the traditional thin ideal, it tends in practice to normalize a highly idealized muscular yet lean physique (Boepple and Thompson, 2014). Tiggemann and Zaccardo (2015) reported that brief exposure to Instagram fitspiration images increased negative affect and body dissatisfaction in women and reduced appearance-related state self-esteem, with these effects mediated by appearance comparison. These findings suggest that even health-oriented images can lead to negative body image outcomes by eliciting social comparison. However, whether actual Korean SOCIAL MEDIA influencer images produce similar effects, and whether post-exposure emotional changes differ by gender, has not been sufficiently investigated.

According to social comparison theory (Festinger, 1954), people evaluate themselves by comparing their bodies and appearance with others. In the SOCIAL MEDIA environment, such comparisons occur more frequently, and upward comparisons with others who possess more idealized physiques may yield body dissatisfaction, negative affect, and lower self-esteem (Barbierik et al., 2023; Fardouly and Vartanian, 2015; Li et al., 2025; McComb and Mills, 2021). SOCIAL MEDIA may intensify these effects relative to traditional media through strong peer presence, abundant visual image exchange, and psychological closeness to content creators, amplifying negative social comparison and peer normative pressure (Perloff, 2014). Influencer-mediated body image exposure must therefore be understood not merely as a media effect, but as a comparison process involving normative body ideals accompanied by psychological proximity. Beyond general body image constructs, this study focuses on subjective body image perception (BIP), which captures the gap between individuals’ perceived current and ideal body shapes. BIP discrepancy has been conceptualized as a core cognitive marker of body-related self-evaluation, with particular relevance to gender-specific ideals — namely, thinness in women and muscularity in men.

Although prior body image research has predominantly focused on women (Çınaroğlu and Yılmazer, 2025; Conboy et al., 2024; Lacroix et al., 2023; Sadık and Yılmaz, 2025), recent studies report that men are also significantly influenced by social media body images (Nimiya et al., 2024). Whereas women tend to strongly internalize the thin ideal (Boepple and Thompson, 2014), men are more exposed to the muscularity ideal, which is a primary source of body image dissatisfaction (Fatt et al., 2019). This gendered difference is captured by the drive for muscularity concept (McCreary and Sasse, 2000), which centers on preoccupation with the discrepancy between one’s current and ideal muscular physique—a gender-specific psychological vulnerability structurally parallel to women’s drive for thinness. Men with higher drive for muscularity are expected to selectively attend to body parts indicative of muscle development (e.g., chest and arms) when viewing social media content, providing a theoretical basis for predicting gender differences in gaze patterns toward specific influencer body parts.

Another key theoretical framework is objectification theory (Fredrickson and Roberts, 1997). According to this theory, women are socialized to internalize their bodies as objects to be evaluated by others’ gaze, developing body surveillance—a tendency to monitor one’s body continuously from an external observer’s perspective. McKinley and Hyde (1996) reported that body surveillance was significantly associated with body shame and disordered eating. Within this framework, the distribution of visual attention toward influencer body images can be interpreted as an indicator of attentional bias—the expression of internalized body surveillance in the processing of external stimuli. The hypervigilance mechanism, whereby attention to threatening or self-evaluative stimuli is amplified under negative affective states, provides a theoretical basis for understanding how affective variables predict gaze patterns toward specific body parts.

Eye tracking enables objective and nonreactive measurement of how individuals process visual stimuli, and its use in body image research has grown rapidly (Couture Bue, 2020; Couture Bue and Harrison, 2020; Scott et al., 2023). Fixation duration and fixation frequency quantify selective attention to specific body parts, reflecting how centrally individuals process a given part during body image evaluation and social comparison. Scott et al. (2023), using an eye-tracking paradigm with an Instagram image array, found that women with lower body satisfaction showed enhanced visual attention toward body-related images. Couture Bue (2020) found that women with higher Instagram use frequency showed increased visual attention toward body-anxiety parts, a relationship mediated by appearance comparison tendency and body dissatisfaction.

However, existing eye-tracking studies have primarily focused on women alone or used artificially constructed stimuli rather than actual SOCIAL MEDIA influencer images. Studies that comparatively analyze the relationships between body image psychological variables and eye-tracking metrics by gender are very scarce. In particular, eye-tracking research utilizing actual Instagram influencer images in an East Asian cultural context such as Korea has rarely been reported. Accordingly, the present study used photographs of Korean sports and health Instagram influencers to examine gender differences in gaze patterns toward specific body parts in healthy adults with normal-range BMI, and to elucidate how BIP, body satisfaction (BS), and negative affect (PANAS) predict these patterns and how these predictive relationships differ by gender.

The specific aims were as follows: (1) to analyze gender differences in AOI-based gaze patterns (fixation duration and fixation frequency); (2) to examine pre-to-post changes in BIP, BS, and PANAS and their gender differences using mixed-design ANOVA; (3) to explore associations between pre-exposure psychological variables and gaze metrics using FDR-corrected correlation analysis; and (4) to construct gender-specific regression models predicting AOI-based gaze patterns from BIP, BS, and PANAS.

## 2 Methods

### 2.1 Study design

This study employed a mixed design, with gender (male/female) as the between-subjects factor and time point (pre-exposure/post-exposure) as the within-subjects factor. Pre-exposure psychological questionnaires (BIP, BS, and PANAS) were administered at least 1 week prior to visual stimulus exposure, and identical questionnaires were administered immediately after exposure. The eye-tracking task was performed during stimulus exposure. This study was approved by the Institutional Review Board of Sungkyunkwan University, and written informed consent was obtained from all participants.

### 2.2 Participants

Participants were 60 healthy Korean adults (30 males, 30 females) residing in Suwon, South Korea, recruited via convenience sampling. Inclusion criteria were: (1) normal BMI (18.5–22.9 kg/m^2^) according to WHO Asia-Pacific standards (World Health Organization, 2000); (2) age ≥ 18 years; (3) normal or soft-contact-lens-corrected vision; (4) no history of mental health diagnoses; and (5) not currently enrolled in a diet or muscle-building program. Participants who wore eyeglasses were instructed to switch to soft contact lenses to ensure eye-tracking accuracy. Participant characteristics and baseline psychological measures are presented in Table 1.

**Table 1:** Participant characteristics and baseline psychological measures.

Variable	Male (*n* = 30) *M* ± SD	Female (*n* = 30) *M* ± SD
Age (years)	23.27 ± 2.80	23.87 ± 2.45
BMI (kg/m^2^)	21.75 ± 1.18	20.64 ± 1.35
BIP: Present figure	3.67 ± 1.09	4.83 ± 1.05
BIP: Ideal figure	4.97 ± 0.93	3.70 ± 0.95
BIP: Subjective figure	2.47 ± 0.68	3.10 ± 0.71
BIP: Discrepancy	−1.30 ± 1.32	1.13 ± 1.14
BS1: Face	2.83 ± 0.65	2.83 ± 0.53
BS2: Hair	2.80 ± 0.92	3.07 ± 0.74
BS3: Skin	2.53 ± 0.86	2.60 ± 0.81
BS4: Lower body	2.73 ± 0.58	2.33 ± 0.84
BS5: Waist and abdomen	2.63 ± 0.72	2.40 ± 0.89
BS6: Upper body	2.67 ± 0.88	2.50 ± 0.73
BS7: Weight	2.73 ± 0.74	2.50 ± 0.90
BS8: Height	2.70 ± 0.95	2.77 ± 0.90
BS9: Overall appearance	2.77 ± 0.68	2.77 ± 0.57
PANAS-N composite	1.68 ± 0.74	1.71 ± 0.81
PANAS-P composite	2.82 ± 0.65	2.65 ± 0.71

M = mean; SD = standard deviation; BIP = body image perception (Stunkard et al., 1983; 1–9 figure scale); BIP Discrepancy = BIP-present − BIP-ideal (negative values indicate desire for greater muscularity; positive values indicate desire for leanness); BS = body satisfaction (Cash, 2000; 1–4 scale, higher = more satisfied); PANAS = positive and negative affect schedule (Watson et al., 1988; 1–5 scale, higher = stronger affect).

No formal *a priori* power analysis was conducted, which is acknowledged as a methodological limitation. *Post hoc* power analysis using G*Power 3.1.9.7 (Faul et al., 2007), assuming a medium effect size (*f* = 0.25), indicated that statistical power to detect the Gender × AOI interaction in the mixed ANOVA was approximately 1 − *β* = 0.72, somewhat below the conventionally recommended threshold of 0.80. For the primary correlation analyses (*n* = 30, observed |*r*| = 0.56, *α* = 0.05), statistical power was high (1 − *β* = 0.94). Exploratory analyses involving multiple AOI comparisons may have had insufficient power, and results of those analyses should be interpreted at an exploratory level.

### 2.3 Measures

#### 2.3.1 Body image perception (BIP)

Subjective body image perception was assessed using the body silhouette scale developed by Stunkard et al. (1983), consisting of nine male and female body figures ranging from very thin to very obese. Participants selected the figure that best represented their current body shape (BIP-present), their ideal body shape (BIP-ideal), and rated their subjective body shape on a 5-point scale (1 = very thin; 5 = very overweight; BIP-subjective).

#### 2.3.2 BIP discrepancy

The BIP discrepancy was calculated as BIP-present minus BIP-ideal. A positive value indicates that the participant perceives their current body as larger than their ideal (i.e., desire to be thinner), whereas a negative value indicates perception of being smaller than ideal (i.e., desire for greater muscularity). Validity and reliability of this scale have been confirmed in Korean adult samples (Jung et al., 2015).

#### 2.3.3 Body satisfaction (BS)

Body satisfaction was measured using nine subscale items adapted from the Body Areas Satisfaction Scale (Cash, 2000) for Korean culture. Items assessed satisfaction with face (BS1), hair (BS2), skin (BS3), lower body (BS4), waist/abdomen (BS5), upper body (BS6), weight (BS7), height (BS8), and overall appearance (BS9), each rated on a 4-point Likert scale (1 = very dissatisfied; 4 = very satisfied). Each item was used independently in analyses (BS1–BS9), and a composite score was computed for exploratory purposes.

#### 2.3.4 Positive and negative affect (PANAS)

Affective state was measured using a brief 10-item version of the Positive and Negative Affect Schedule (PANAS) (Watson et al., 1988). The scale comprised five negative affect items (PANAS1 = scared, PANAS2 = upset, PANAS3 = irritable, PANAS4 = afraid, PANAS5 = distressed) and five positive affect items (PANAS6–PANAS10), each rated on a 5-point scale (1 = not at all; 5 = extremely). Composite scores (PANAS-N and PANAS-P) were the means of the respective five items. The Korean version of the PANAS has demonstrated adequate reliability and construct validity in Korean adult samples (Lee et al., 2003).

It should be noted that the present study did not include direct measures of body surveillance (McKinley and Hyde, 1996), body shame, the Drive for Muscularity Scale (DMS; McCreary and Sasse, 2000), or social media use frequency. Objectification theory (Fredrickson and Roberts, 1997) and social comparison theory (Festinger, 1954) are employed as theoretical frameworks for interpreting gaze patterns, not as constructs directly operationalized by the current measurement battery. Similarly, references to drive for muscularity in the discussion reflect theoretical interpretation of BIP discrepancy data; the DMS was not administered. These omissions are acknowledged as limitations that restrict the directness of theoretical inference and are addressed further in Section 4.4.

### 2.4 Eye-tracking procedure

#### 2.4.1 Environmental setup

All eye-tracking procedures were conducted in a neutral office environment. Participants were seated at a monitor separated from the researcher and research assistants by a partition. Research assistants were all male and wore loose, solid-colored clothing with no text, avoiding cosmetics and perfume. Participants were also asked to remove their makeup and avoid wearing perfume (Couture Bue and Harrison, 2020).

#### 2.4.2 Visual stimuli

Visual stimuli consisted of 15 photographs from Korean Instagram sports and health influencers ranked within the top 15 in their respective category on Korean Instagram; each participant was exposed only to same-sex influencer images. Each image was presented for 20 s (total exposure: 300 s). Schematic illustrations representing all 30 stimulus images (15 female, 15 male) used in the experiment are provided in Supplementary Figures S1, S2, respectively; these illustrations preserve the body posture, contour, and AOI-boundary characteristics of the original photographs while omitting photographic and facial detail for copyright and privacy protection. Eye movements were recorded using a Tobii Pro Fusion eye tracker (Tobii Technology AB, Sweden) at 120 Hz. The monitor was 22 inches (display area: 478.7 × 260.3 mm), with a 16:9 aspect ratio, 1920 × 1,080 resolution, and 60 Hz refresh rate. Viewing distance was maintained at 60–70 cm. Prior to recording, all participants completed a 9-point calibration.

Influencer selection criteria included: (1) ≥ 10,000 followers; (2) documented activity within the previous 6 months; and (3) accounts primarily featuring posts with clearly visible body images rather than explicit commercial advertising. Stimulus images were standardized to 1920 × 1,080 pixels; images with simple backgrounds (indoor or solid color) were prioritized. Clothing was limited to sportswear categories (functional tops, leggings, shorts) sufficient to reveal body contours. Overexposed or excessively dark images were excluded.

#### 2.4.3 Area of interest (AOI) definitions

**Figure 1:**
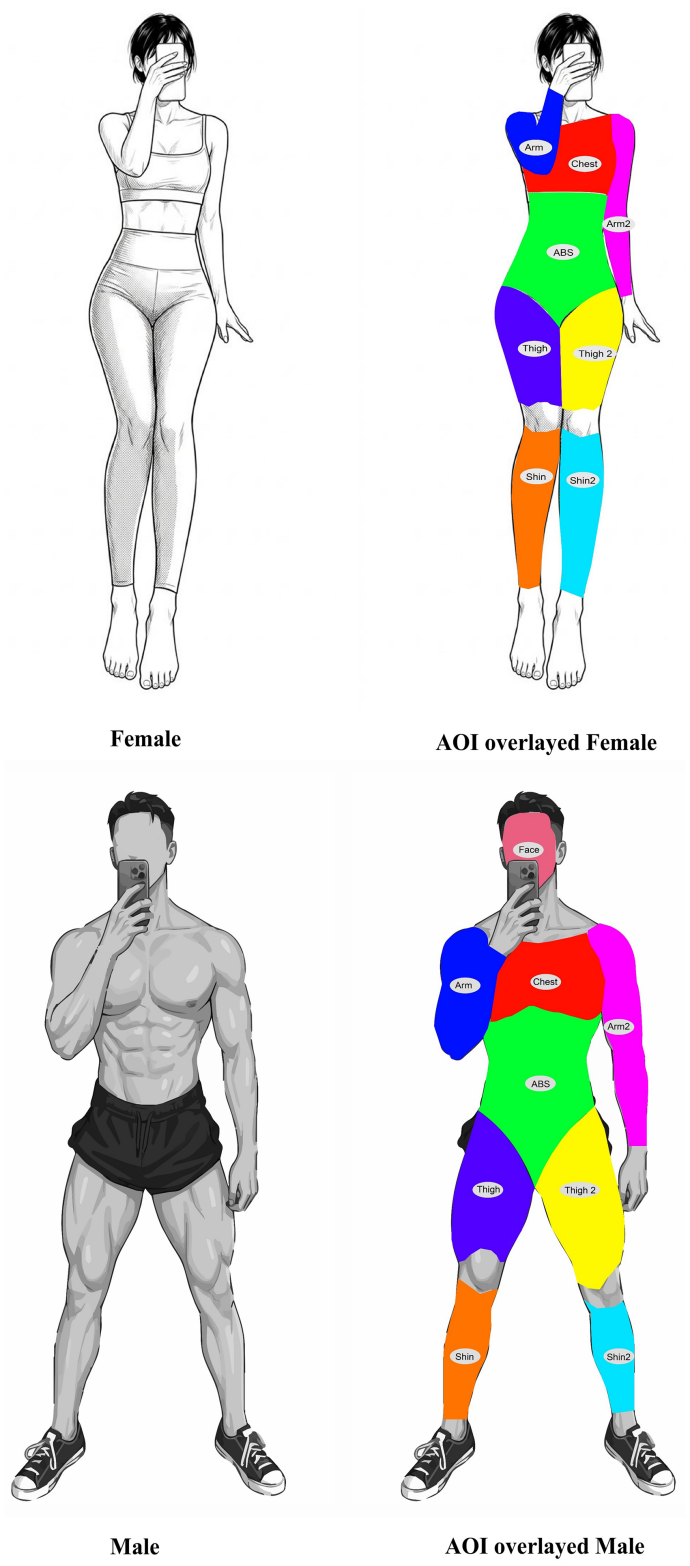
Schematic illustration of the areas of interest (AOIs) used in the eye-tracking analysis, depicting the body contour and posture of the stimulus photographs for a female (top) and a male (bottom) figure. A total of nine AOIs were defined: Face (crown to chin), Chest (below clavicle to upper abdomen), Arm (shoulder joint to wrist), Abdomen (upper abdomen to lower pelvis), Hip (pelvis to proximal thigh), Thigh (proximal thigh to above knee), Shin (anterior lower leg), Calf (posterior lower leg), and Trunk back (posterior upper torso). Six AOIs visible from the anterior view (Face, Chest, Arm, Abdomen, Thigh, and Shin) are color-coded in this figure. Although the hip contour is partly visible from the anterior view, its boundary overlaps with the thigh and abdomen, making it difficult to delineate consistently; the Hip AOI was therefore defined based on the lateral and posterior contours and is not shown here. The remaining two AOIs (Calf, Trunk back) were defined on the posterior view and are likewise not shown here. AOI boundaries were applied consistently across all 30 stimulus photographs used in the study (15 female, 15 male); schematic illustrations of the full stimulus set are provided in Supplementary Figures S1, S2. Schematic illustrations, rather than the original influencer photographs, are presented here to avoid copyright and privacy concerns, while preserving the posture and AOI-boundary definitions of the original images.

Nine AOIs were defined on each image using Tobii Pro Lab software: Face (crown to chin), Chest (below clavicle to upper abdomen), Arm (shoulder joint to wrist), Abdomen (upper abdomen to lower pelvis), Hip (pelvis to proximal thigh), Thigh (proximal thigh to above knee), Shin (below knee to ankle), Calf (posterior lower leg), and Trunk back (posterior upper torso). Because the anterior boundary of the hip overlapped with the abdomen and thigh, the Hip AOI was delineated using the lateral and posterior body contour rather than the anterior view. An example of the AOI labeling is presented in Figure 1. AOI boundaries were delineated manually by the same researcher across all images to ensure consistency. The primary gaze metrics were fixation duration ratio (% of total fixation time) and fixation frequency ratio (% of total fixations) for each AOI.

### 2.5 Statistical analysis

All statistical analyses were conducted using self-developed Python scripts (version ≥ 3.9), with primary libraries including pandas, NumPy, SciPy, statsmodels, and pingouin. Descriptive statistics are reported as M ± SD.

**Figure 2:**
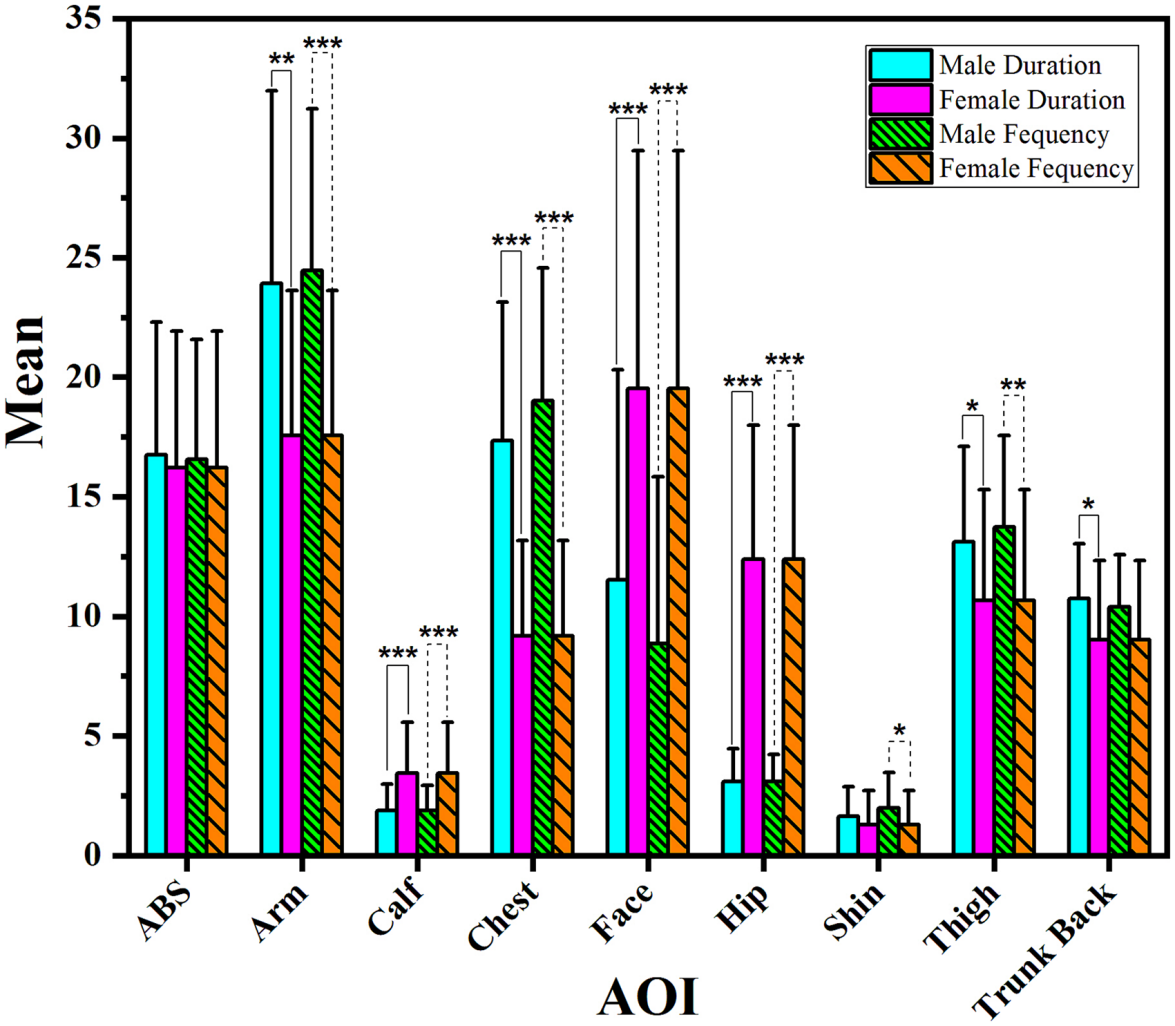
Mean proportions of fixation duration and fixation frequency by AOI and gender. Bars represent the mean percentage of total fixation time (solid fill) and total fixation count (hatched fill) allocated to each of the nine AOIs (Abdomen, Arm, Calf, Chest, Face, Hip, Shin, Thigh, Trunk back) while participants viewed same-sex influencer body images. Error bars indicate one standard deviation. Asterisks denote statistically significant gender differences based on independent-samples *t*-tests. *p < 0.05, **p < 0.01, ***p < 0.001.

Gender differences in AOI-based gaze metrics were assessed using independent-samples *t*-tests (Figure 2). Normality within each cell was verified using Kolmogorov–Smirnov and Shapiro–Wilk tests, and homogeneity of variance was assessed using Levene’s test; violations resulted in automatic application of Welch’s t-test or the Mann–Whitney U test. Main effects of AOI and gender and their interaction were analyzed using a 2 (gender) × 9 (AOI) mixed-design ANOVA; sphericity violations were corrected using the Greenhouse–Geisser method. Effect sizes are reported as partial *η*^2^ (Table 2).

**Table 2:** Mixed ANOVA for the effects of gender and AOI on fixation duration and fixation frequency.

Outcome	Effect	*F*	df	*p* (GG-corrected)	Partial *η*^2^	Sig.
Fixation duration	Gender	1.87	1, 58	0.177	0.031	ns
	AOI	79.98	3.44, 249.7	<0.001	0.580	***
	Gender × AOI	16.61	3.44, 249.7	<0.001	0.223	***
Fixation frequency	Gender	1.85	1, 58	0.179	0.031	ns
	AOI	89.77	3.25, 232.6	< 0.001	0.608	***
	Gender × AOI	25.15	3.25, 232.6	< 0.001	0.303	***

*F* statistics are reported with Greenhouse–Geisser-corrected degrees of freedom and p values for the main effects of Gender (male vs. female) and AOI (nine body parts) and their interaction (Gender × AOI) from 2 × 9 mixed ANOVAs. Greenhouse–Geisser correction was applied because Mauchly’s tests indicated violations of sphericity (*W* = 0.0008, *p* < 0.001). Partial *η*^2^ is the measure of effect size. ns = not significant (*p* ≥ 0.05); ***p < 0.001.

Pre-to-post changes in BIP, BS, and PANAS and gender differences were examined using 2 (gender) × 2 (time) mixed-design ANOVAs (Tables 3, 4). When interaction effects were significant, simple effects were analyzed using paired-samples t-tests (Cohen’s dz) and independent-samples *t*-tests (Cohen’s *d*), supplemented by Bonferroni and Sidak *post hoc* tests. Third, associations between pre-exposure psychological variables and AOI-based gaze metrics were explored using Pearson correlation analysis with Benjamini and Hochberg (1995) FDR correction applied independently within each subset defined by gender and metric type (fixation duration/frequency; Tables 5–8); q_FDR < 0.05 was the threshold for significance after correction. Finally, variable pairs showing significant FDR-corrected correlations were entered into simple linear, multiple linear, or hierarchical regression models using statsmodels.api.OLS (Table 9); multicollinearity was assessed using VIF and tolerance, with VIF < 5 indicating acceptable levels. The significance level was *α* = 0.05.

**Table 3:** Mixed ANOVA results for body image perception, body satisfaction, and affect measures.

Measure	Effect	*F*	df (GG-corrected)	*p*	Partial *η*^2^_p_	Sig.
BIP: Present figure	Gender	17.10	1, 58	<0.001	0.228	***
	Time	1.27	1, 58	0.264	0.022	ns
	Time × Gender	0.14	1, 58	0.708	0.002	ns
BIP: Ideal figure	Gender	46.11	1, 58	<0.001	0.443	***
	Time	0.99	1, 58	0.325	0.017	ns
	Time × Gender	0.18	1, 58	0.672	0.003	ns
BIP: Subjective figure	Gender	12.95	1, 58	<0.001	0.183	***
	Time	0.33	1, 58	0.570	0.006	ns
	Time × Gender	0.33	1, 58	0.570	0.006	ns
BIP: Discrepancy	Gender	78.09	1, 58	<0.001	0.574	***
	Time	0.01	1, 58	0.903	0.000	ns
	Time × Gender	0.01	1, 58	0.903	0.000	ns
BS4: Lower body	Gender	4.95	1, 58	0.030	0.079	*
	Time	0.59	1, 58	0.447	0.010	ns
	Time × Gender	0.07	1, 58	0.799	0.001	ns
PANAS-N composite	Gender	0.13	1, 58	0.722	0.002	ns
	Time	10.99	1, 58	0.002	0.159	**
	Time × Gender	0.71	1, 58	0.402	0.012	ns
PANAS-P composite	Gender	2.15	1, 58	0.148	0.036	ns
	Time	0.49	1, 58	0.485	0.008	ns
	Time × Gender	0.75	1, 58	0.389	0.013	ns
PANAS2 (upset)	Gender	0.17	1, 58	0.677	0.003	ns
	Time	6.17	1, 58	0.016	0.096	*
	Time × Gender	0.05	1, 58	0.822	0.001	ns
PANAS3 (irritable)	Gender	0.02	1, 58	0.902	0.000	ns
	Time	5.69	1, 58	0.020	0.089	*
	Time × Gender	0.36	1, 58	0.553	0.006	ns
PANAS5 (distressed)	Gender	0.68	1, 58	0.415	0.012	ns
	Time	7.35	1, 58	0.009	0.113	**
	Time × Gender	0.11	1, 58	0.736	0.002	ns

The 2 (Gender: between-subjects) × 2 (Time: pre/post exposure; within-subjects) mixed ANOVA was conducted for each measure. *N* = 60 (males *n* = 30, females *n* = 30). Only measures showing at least one significant effect are reported. GG = Greenhouse–Geisser correction applied where Mauchly’s test indicated violation of sphericity. BIP = Body Image Perception (Stunkard et al., 1983); BS = Body Satisfaction (Cash, 2000); PANAS = Positive and Negative Affect Schedule (Watson et al., 1988). PANAS2 = upset; PANAS3 = irritable; PANAS5 = distressed. *η*^2^_p_ = partial eta-squared.

*p < 0.05, **p < 0.01, ***p < 0.001; ns, not significant.

**Table 4:** Descriptive statistics for body image perception, body satisfaction, and affect by gender and time.

Measure	Subscale/item	Time	Male (*n* = 30) *M* ± SD	Female (*n* = 30) *M* ± SD
BIP	Present figure	Pre	3.67 ± 1.09	4.83 ± 1.05
		Post	3.60 ± 1.10	4.70 ± 1.21
	Ideal figure	Pre	4.97 ± 0.93	3.70 ± 0.95
		Post	4.90 ± 0.76	3.53 ± 0.86
	Subjective figure	Pre	2.47 ± 0.68	3.10 ± 0.71
		Post	2.43 ± 0.73	3.10 ± 0.71
	Discrepancy	Pre	−1.30 ± 1.32	1.13 ± 1.14
		Post	−1.30 ± 1.24	1.17 ± 1.09
BS	Lower body (BS4)	Pre	2.87 ± 0.73	3.37 ± 0.96
		Post	2.80 ± 0.71	3.30 ± 0.99
PANAS	Negative affect composite	Pre	1.68 ± 0.74	1.71 ± 0.81
		Post	1.47 ± 0.68	1.58 ± 0.89
	Positive affect composite	Pre	2.57 ± 0.82	2.77 ± 0.94
		Post	2.60 ± 0.93	2.67 ± 0.88
	PANAS2 (upset)	Pre	1.60 ± 0.86	1.67 ± 0.88
		Post	1.40 ± 0.72	1.50 ± 0.82
	PANAS3 (irritable)	Pre	2.00 ± 1.11	1.97 ± 1.19
		Post	1.67 ± 0.99	1.77 ± 1.22
	PANAS5 (distressed)	Pre	1.70 ± 0.99	1.87 ± 1.04
		Post	1.40 ± 0.81	1.63 ± 1.19

BIP = Body Image Perception; BS = Body Satisfaction; PANAS = Positive and Negative Affect Schedule. Only measures with at least one significant ANOVA effect are presented. Pre-exposure measurements were taken at least 1 week before the visual exposure session. Post-exposure measurements were taken immediately after 300 s of same-sex influencer body image exposure. PANAS2 = upset; PANAS3 = irritable; PANAS5 = distressed.

**Table 5:** Pearson correlations between pre-exposure psychological variables and AOI fixation duration in the male group (ordered by q_FDR).

Psychological variable	AOI	*r*	*p*	q_FDR	FDR sig.
BIP discrepancy (current − ideal)	Chest	−0.563	0.001	0.028	†
Subjective BIP	Chest	−0.557	0.001	0.030	†
BS5 (waist/abdominal satisfaction)	Shin	+0.491	0.006	0.084	
BIP discrepancy (current − ideal)	Face	+0.486	0.006	0.090	
BS2 (hair satisfaction)	Shin	+0.484	0.007	0.092	
BIP-present (current figure rating)	Chest	−0.479	0.008	0.093	
Subjective BIP	Face	+0.461	0.010	0.115	
BS8 (height satisfaction)	Trunk back	+0.456	0.011	0.118	
BS5 (waist/abdominal satisfaction)	Calf	+0.453	0.012	0.121	
BS7 (weight satisfaction)	Chest	−0.424	0.020	0.175	
BS2 (hair satisfaction)	Chest	−0.416	0.022	0.190	
BIP-ideal (ideal figure rating)	Face	−0.412	0.024	0.197	
PANAS3 (irritable)	Calf	+0.393	0.032	0.235	
BS4 (lower body satisfaction)	Shin	+0.367	0.046	0.298	
BS3 (skin satisfaction)	Face	−0.364	0.048	0.299	
PANAS7 (positive affect)	Shin	+0.361	0.050	0.305	

Values are Pearson *r* with uncorrected *p*-values and Benjamini–Hochberg FDR-corrected *q*-values. AOI = area of interest; BIP discrepancy = BIP-present − BIP-ideal (negative = desire for muscularity); Subjective BIP = 1–9 figure rating (higher = more overweight perception); BS = Body Satisfaction subscale; PANAS = Positive and Negative Affect Schedule items. PANAS3 = irritable. *n* = 30. ^†^q_FDR < 0.05 (FDR-corrected significant).

**Table 6:** Pearson correlations between psychological change scores (*Δ* = post − pre) and AOI fixation frequency in the male group (ordered by q_FDR).

Psychological variable	AOI	*r*	*p*	q_FDR	FDR sig.
ΔBS8 (height satisfaction change)	Shin	+0.55	0.001	0.029	†
ΔBS7 (weight satisfaction change)	Chest	+0.46	0.012	0.186	
ΔPANAS-N (negative affect composite)	Abdomen	−0.43	0.019	0.260	
ΔPANAS2 (upset)	Abdomen	−0.40	0.028	0.316	
ΔSubjective BIP	Trunk back	−0.40	0.030	0.316	
ΔPANAS2 (upset)	Shin	+0.39	0.032	0.318	
ΔBS8 (height satisfaction change)	Calf	+0.38	0.039	0.354	
ΔBS6 (upper body satisfaction change)	Chest	+0.38	0.040	0.354	
ΔBS2 (hair satisfaction change)	Thigh	−0.37	0.043	0.373	

Δ = post-exposure minus pre-exposure change score. AOI = area of interest; BS = Body Satisfaction subscale; PANAS-N = negative affect composite; Subjective BIP = 1–9 figure rating. PANAS2 = upset. *n* = 30. ^†^q_FDR < 0.05 (FDR-corrected significant).

**Table 7:** Pearson correlations between pre-exposure psychological variables and AOI fixation duration in the female group (ordered by q_FDR).

Psychological variable	AOI	*r*	*p*	q_FDR	FDR sig.
PANAS2 (upset)	Trunk back	+0.535	0.002	0.039	†
PANAS2 (upset)	Chest	+0.524	0.003	0.042	†
BS2 (hair satisfaction)	Thigh	+0.485	0.007	0.066	
PANAS2 (upset)	Thigh	−0.459	0.011	0.095	
PANAS-N composite	Thigh	−0.450	0.013	0.105	
PANAS6 (positive affect)	Shin	+0.444	0.014	0.108	
PANAS7 (positive affect)	Thigh	+0.443	0.014	0.109	
PANAS-P composite	Shin	+0.436	0.016	0.116	
PANAS9 (positive affect)	Shin	+0.425	0.019	0.128	
Subjective BIP	Calf	+0.411	0.024	0.148	
PANAS-P composite	Thigh	+0.402	0.028	0.161	
PANAS5 (distressed)	Thigh	−0.402	0.028	0.161	
PANAS9 (positive affect)	Hip	+0.382	0.037	0.198	
BS7 (weight satisfaction)	Thigh	+0.382	0.038	0.199	
PANAS3 (irritable)	Thigh	−0.373	0.042	0.214	
BS2 (hair satisfaction)	Face	−0.373	0.042	0.214	

Values are Pearson *r* with uncorrected *p*-values and Benjamini–Hochberg FDR-corrected *q*-values. AOI = area of interest; BS = Body Satisfaction subscale; PANAS-N = negative affect composite; PANAS-P = positive affect composite; Subjective BIP = 1–9 figure rating. PANAS item labels: PANAS2 = upset; PANAS3 = irritable; PANAS5 = distressed; PANAS6, PANAS7, PANAS9 = positive affect items. *n* = 30. ^†^q_FDR < 0.05 (FDR-corrected significant).

**Table 8:** Pearson correlations between pre-exposure psychological variables and AOI fixation frequency in the female group (ordered by q_FDR).

Psychological variable	AOI	*r*	*p*	q_FDR	FDR sig.
BS6 (upper body satisfaction)	Thigh	+0.564	0.001	0.023	†
PANAS1 (scared)	Thigh	−0.529	0.003	0.042	†
BS2 (hair satisfaction)	Thigh	+0.508	0.004	0.051	
BS9 (overall appearance)	Trunk back	+0.490	0.006	0.060	
PANAS8 (positive affect)	Hip	−0.469	0.009	0.078	
PANAS9 (positive affect)	Chest	−0.464	0.010	0.083	
PANAS1 (scared)	Face	+0.442	0.014	0.110	
BS6 (upper body satisfaction)	Chest	−0.439	0.015	0.111	
PANAS4 (negative affect)	Thigh	−0.434	0.017	0.116	
PANAS8 (positive affect)	Arm	+0.433	0.017	0.116	
PANAS3 (irritable)	Calf	−0.431	0.017	0.117	
PANAS-N composite	Thigh	−0.431	0.018	0.117	
BS6 (upper body satisfaction)	Hip	+0.415	0.023	0.135	
PANAS-N composite	Face	+0.398	0.029	0.162	
PANAS4 (negative affect)	Face	+0.396	0.030	0.162	
BS1 (face satisfaction)	Trunk back	+0.395	0.031	0.163	
PANAS5 (distressed)	Calf	−0.389	0.034	0.174	
BS7 (weight satisfaction)	Thigh	+0.375	0.041	0.203	
BS3 (skin satisfaction)	Trunk back	+0.367	0.046	0.219	

Values are Pearson *r* with uncorrected *p*-values and Benjamini–Hochberg FDR-corrected *q*-values. AOI = area of interest; BS = Body Satisfaction subscale; PANAS-N = negative affect composite; Subjective BIP = 1–9 figure rating. PANAS item labels: PANAS1 = scared; PANAS2 = upset; PANAS3 = irritable; PANAS4 = afraid; PANAS5 = distressed; PANAS8, PANAS9 = positive affect items. *n* = 30. ^†^q_FDR < 0.05 (FDR-corrected significant).

**Table 9:** Regression models predicting AOI-based gaze metrics from psychological variables.

Group	Predictor → Outcome	*B*	SE	*t*	*p*	*R* ^2^	Adj. R^2^	*F*(df₁, df₂)
Female group (*n* = 30)								
Female	PANAS2 (upset) → Chest AOI fixation duration	2.353	0.722	3.259	0.003**	0.275	0.249	10.62(1, 28)**
	PANAS2 (upset) → Trunk back AOI fixation duration	1.999	0.596	3.353	0.002**	0.286	0.261	11.24(1, 28)**
	Overall model: BS6 + PANAS1 → Thigh AOI fixation frequency	—	—	—	0.002**	0.378	0.332	8.22(2, 27)**
	BS6 (upper body satisfaction)	2.588	1.253	2.065	0.049*	—	—	—
	PANAS1 (scared)	−1.602	0.987	−1.623	0.116	—	—	—
Male group (*n* = 30)								
Male	BS5 (waist/abdominal satisfaction) → Calf AOI fixation frequency	0.825	0.229	3.604	0.001**	0.317	0.293	12.99(1, 28)**
	Shin AOI fixation frequency → ΔBS8 (height satisfaction change)^†^	0.196	0.045	4.404	< 0.001***	0.409	0.388	19.40(1, 28)***
	Hierarchical model: Chest AOI fixation duration	—	—	—	—	—	—	—
	Step 1: Subjective BIP	—	—	—	0.001**	0.310	0.285	12.58(1, 28)**
	Step 2: + BIP discrepancy (current − ideal)	—	—	—	0.002**	0.378	0.332	8.20(2, 27)**

B = unstandardized regression coefficient; SE = standard error; *R*^2^ = coefficient of determination; Adj. *R*^2^ = adjusted *R*^2^. All models are ordinary least squares regressions. Rows indented with spaces represent individual predictor coefficients within a multiple or hierarchical model; overall model fit statistics appear in the model-header row. PANAS2 = upset; PANAS1 = scared (Watson et al., 1988); BS5 = waist/abdominal satisfaction; BS6 = upper body satisfaction; BS8 = height satisfaction; Subjective BIP = 1–9 figure rating (higher = more overweight perception); BIP discrepancy = BIP-present − BIP-ideal (negative = drive for muscularity). All VIF < 5 (multicollinearity acceptable).

^†^Exploratory model in which gaze frequency predicts post-minus-pre satisfaction change. *p < 0.05, **p < 0.01, ***p < 0.001.

## 3 Results

### 3.1 Participant characteristics

Participant characteristics and pre-exposure psychological measures are presented in Table 1. Males (*M* = 4.97) desired a larger ideal body than females (*M* = 3.70) on BIP-ideal, whereas females (*M* = 4.83) perceived their current body as larger than males (*M* = 3.67). BIP discrepancy was negative for males (*M* = −1.30) and positive for females (*M* = 1.13), confirming that males perceived their current body as smaller than ideal (desire for greater muscularity) whereas females perceived their current body as larger than ideal (desire for leanness).

### 3.2 Gender differences in AOI-based gaze metrics

Descriptive statistics and gender comparison results are presented in Table 10 and Figure 2. The 2 × 9 mixed ANOVA revealed significant main effects of AOI for both fixation duration and fixation frequency (*F* = 79.98 and *F* = 89.77, respectively; both *p* < 0.001; partial *η*^2^ > 0.58) and significant Gender × AOI interaction effects (*F* = 16.61 and *F* = 25.15, respectively; both *p* < 0.001; partial *η*^2^ > 0.22; Table 2). The gender main effect was not significant (*p* > 0.05).

**Table 10:** Descriptive statistics of gaze metrics by area of interest (AOI) and gender.

AOI	Metric	Male (*n* = 30) *M* ± SD	Female (*n* = 30) *M* ± SD
Abdomen	Duration (%)	16.75 ± 5.58	16.22 ± 5.71
	Frequency (%)	16.57 ± 5.01	16.49 ± 5.32
Arm	Duration (%)	23.91 ± 8.06	17.57 ± 6.07
	Frequency (%)	24.47 ± 6.76	18.63 ± 5.99
Calf	Duration (%)	1.88 ± 1.12	3.45 ± 2.12
	Frequency (%)	1.87 ± 1.05	3.93 ± 2.12
Chest	Duration (%)	17.36 ± 5.77	9.20 ± 3.97
	Frequency (%)	19.02 ± 5.54	9.95 ± 3.89
Face	Duration (%)	11.54 ± 8.78	19.53 ± 9.94
	Frequency (%)	8.86 ± 6.99	15.45 ± 8.56
Hip	Duration (%)	3.08 ± 1.38	12.39 ± 5.60
	Frequency (%)	3.09 ± 1.12	12.55 ± 4.81
Shin	Duration (%)	1.63 ± 1.25	1.29 ± 1.42
	Frequency (%)	2.00 ± 1.47	1.27 ± 1.38
Thigh	Duration (%)	13.12 ± 3.97	10.66 ± 4.65
	Frequency (%)	13.73 ± 3.82	11.51 ± 4.90
Trunk back	Duration (%)	10.74 ± 2.29	9.03 ± 3.30
	Frequency (%)	10.39 ± 2.18	9.55 ± 3.02

AOI = area of interest. Gaze metrics (Duration and Frequency) are expressed as the percentage of total fixation time and total fixation count, respectively, allocated to each AOI across all 15 influencer images.

Males showed significantly higher fixation proportions on the chest (duration: 17.36% vs. 9.20%; frequency: 19.02% vs. 9.95%) and arm (duration: 23.91% vs. 17.57%; frequency: 24.47% vs. 18.63%) relative to females. Females showed significantly higher fixation proportions on the face (duration: 19.53% vs. 11.54%; frequency: 15.45% vs. 8.86%) and hip (duration: 12.39% vs. 3.08%; frequency: 12.55% vs. 3.09%) relative to males.

### 3.3 Pre-to-post changes in body image and affect variables

Mixed ANOVA results for BIP, BS, and PANAS variables are presented in Tables 3, 4. All four BIP variables showed significant gender main effects (all *p* < 0.001; partial *η*^2^ = 0.183–0.574), indicating systematic baseline differences between groups. Time main effects and Gender × Time interactions were all non-significant for BIP variables (*p* > 0.05). Among BS subscales, only lower body satisfaction (BS4) showed a significant gender main effect [*F*(1, 58) = 4.95, *p* = 0.030, partial *η*^2^ = 0.079].

The composite negative affect score (PANAS-N) showed a significant time main effect [*F*(1, 58) = 10.99, *p* = 0.002, partial *η*^2^ = 0.159], indicating a significant decrease following exposure. Significant post-exposure reductions were also observed for PANAS2 (upset) [*F*(1, 58) = 6.17, *p* = 0.016, partial *η*^2^ = 0.096], PANAS3 (irritable) [*F*(1, 58) = 5.69, *p* = 0.020, partial *η*^2^ = 0.089], and PANAS5 (distressed) [*F*(1, 58) = 7.35, *p* = 0.009, partial *η*^2^ = 0.113]. The composite positive affect score (PANAS-P) showed no significant effects (*p* > 0.05). Gender × Time interaction effects were non-significant for all PANAS variables.

### 3.4 FDR-corrected correlations between psychological variables and gaze metrics

#### 3.4.1 Male group

In the male group, BIP discrepancy was significantly and negatively correlated with chest AOI fixation duration after FDR correction (*r* = −0.563, *p* = 0.001, q_FDR = 0.028), and subjective BIP was also significantly and negatively correlated with chest fixation duration (*r* = −0.557, *p* = 0.001, q_FDR = 0.030; Table 5), indicating that greater drive for muscularity was associated with longer fixation on the influencer’s chest. Change in height satisfaction (ΔBS8) was significantly and positively correlated with shin AOI fixation frequency (*r* = +0.55, *p* = 0.001, q_FDR = 0.029; Table 6).

#### 3.4.2 Female group

In the female group, PANAS2 (upset) was significantly and positively correlated with fixation duration on the trunk back AOI (*r* = +0.535, *p* = 0.002, q_FDR = 0.039) and the chest AOI (*r* = +0.524, *p* = 0.003, q_FDR = 0.042) after FDR correction (Table 7), indicating that women with higher pre-exposure upset levels tended to fixate longer on the influencer’s chest and trunk back. For fixation frequency, upper body satisfaction (BS6) was significantly and positively correlated with thigh AOI fixation frequency (*r* = +0.564, *p* = 0.001, q_FDR = 0.023), and PANAS1 (scared) was significantly and negatively correlated with thigh AOI fixation frequency (*r* = −0.529, *p* = 0.003, q_FDR = 0.042; Table 8).

### 3.5 Regression analyses

Regression analyses were conducted for variable pairs showing significant FDR-corrected correlations; all models passed multicollinearity diagnostics (VIF < 5; Table 9).

#### 3.5.1 Female group

PANAS2 (upset) significantly predicted chest AOI fixation duration (*B* = 2.353, SE = 0.722, *t* = 3.259, *p* = 0.003, *R*^2^ = 0.275) and trunk back fixation duration (*B* = 1.999, SE = 0.596, *t* = 3.353, *p* = 0.002, *R*^2^ = 0.286), indicating that a one-unit increase in PANAS2 was associated with approximately 2.35 and 2.00% increases in fixation duration on the chest and trunk back, respectively. A multiple regression model with BS6 and PANAS1 predicting thigh fixation frequency was significant [*R*^2^ = 0.378, *F*(2, 27) = 8.22, *p* = 0.002]; BS6 was a significant individual predictor (*B* = 2.588, *t* = 2.065, *p* = 0.049), while PANAS1 was not (*B* = −1.602, *p* = 0.116).

#### 3.5.2 Male group

Abdominal satisfaction (BS5) significantly predicted calf fixation frequency (*B* = 0.825, SE = 0.229, *t* = 3.604, *p* = 0.001, *R*^2^ = 0.317). In an exploratory model, shin fixation frequency significantly predicted post-exposure height satisfaction change (ΔBS8; *B* = 0.196, SE = 0.045, *t* = 4.404, *p* < 0.001, *R*^2^ = 0.409). Hierarchical regression predicting chest fixation duration yielded a significant model when subjective BIP was entered in Step 1 [*R*^2^ = 0.310, *F*(1, 28) = 12.58, *p* = 0.001]; the model remained significant when BIP discrepancy was added in Step 2 [*R*^2^ = 0.378, *F*(2, 27) = 8.20, *p* = 0.002].

## 4 Discussion

This study measured visual attention patterns toward Korean Instagram sports and health influencer body images using eye tracking in healthy adults with normal-range BMI, and comprehensively analyzed gender differences in association with body image psychological variables (BIP, BS, and PANAS). The following sections discuss the primary findings in relation to prior research and consider their theoretical and practical implications.

### 4.1 Gender differences in AOI-based gaze metrics

One of the most consistent findings in this study was that AOI-based gaze patterns toward influencer body images differed significantly by gender. Males showed higher fixation proportions on the chest and arm parts, whereas females concentrated more fixation on the face and hip parts. These results suggest that males and females activate different psychological mechanisms when processing influencer body images.

The male pattern of gaze concentrated on the chest and arm parts is interpreted as reflecting a gender-specific attentional orientation associated with drive for muscularity (McCreary and Sasse, 2000). The male body ideal centers on the acquisition of a muscular rather than thin physique, and the chest and arm parts of an influencer represent canonical visual indicators of muscular development. Males with stronger drive for muscularity are more likely to selectively allocate attentional resources to body parts that can be compared with their ideal physique. This is consistent with the social comparison theory principle that the characteristics of an upward comparison target determine the dimension of comparison (Festinger, 1954), and is further supported by the correlation and regression findings of this study (BIP discrepancy → chest fixation duration). That is, for males, the influencer’s chest and arm parts are selectively processed not simply because they are salient, but because they serve as comparison referents carrying information directly relevant to their body-related goals.

The high fixation proportion on the face part in females requires multilayered theoretical interpretation. First, the central role of the face in Korean women’s appearance evaluation must be considered. Studies by Kim et al. (2014) and Jung and Lee (2006) reported that Korean women’s face consciousness in appearance evaluation is relatively stronger than in Western cultures, which may reflect a tendency among Korean women to process the influencer’s face as the primary object of appearance comparison. Second, from the perspective of objectification theory (Fredrickson and Roberts, 1997), women are socialized to internalize body surveillance—the continuous monitoring of their bodies from an external observer’s perspective (McKinley and Hyde, 1996). This body surveillance tendency may manifest as preferential attention to socially evaluated parts (i.e., the face) when observing others’ bodies. In other words, women’s face-focused gaze may not reflect personal preference but rather the projection of gendered self-objectification processes onto the processing of external stimuli. This interpretation pertains to cultural factors and requires verification through cross-cultural comparative research.

The high fixation proportion on the hip part observed in females (females: 12.39% vs. males: 3.08%) is also noteworthy. This may reflect the emergence of the hip as an important visual indicator of health and physical fitness in Korean sports and health influencer culture. Within the shift in fitspiration content toward the ideal of “strong is the new skinny,” female users may be using the hip as a benchmark for evaluating muscle development and overall physique. Accordingly, the gender differences in gaze patterns toward influencer body images can be understood not merely as reflections of individual interests but as the complex product of gendered body ideals, levels of self-objectification, and cultural aesthetic norms (Boepple and Thompson, 2014; Fatt et al., 2019).

### 4.2 Pre-to-post exposure changes in psychological variables

Mixed ANOVA results showed no significant pre-to-post changes in BIP or body satisfaction variables. This suggests that a 300-s brief exposure may be insufficient to produce immediate changes in cognitive-evaluative constructs such as body image perception and body satisfaction (Gow et al., 2025). Body image perception is understood to rest on a relatively stable self-schema that changes gradually through repeated and prolonged exposure rather than through a single brief episode, consistent with the relative stability of body image self-schemas (De Vignemont, 2010). However, body satisfaction may be amenable to immediate improvement even with brief exposure (Jiménez-García et al., 2025), and outcomes may vary as a function of stimulus characteristics (e.g., degree of sexualization) and individual differences (e.g., media addiction) (Guizzo et al., 2021); future research should examine these moderating variables.

In contrast, the significant post-exposure decreases in the composite negative affect score (PANAS-N) and several individual negative affect items (upset, irritable, distressed) carry important theoretical implications. These findings run counter to the pattern of “fitspiration exposure → increased negative affect” observed in prior research (Tiggemann and Zaccardo, 2015). Three alternative mechanisms may explain this discrepant result.

First, the possibility of an upward assimilation effect warrants consideration. Social comparison theory allows for two different psychological outcomes from the same upward comparison stimulus. Under upward contrast, the comparison target is perceived as too dissimilar to the self, leading to decreased self-evaluation; under upward assimilation, the comparison target is perceived as an attainable goal, eliciting inspiration or motivation (Festinger, 1954). Korean Instagram sports and health influencers used in this study, unlike fashion models or celebrities, may be perceived as “attainable role models” who share specific exercise routines and diets on actual SOCIAL MEDIA platforms. This perception may have triggered inspirational rather than threatening processing, thereby attenuating negative affect.

Second, methodological differences from the fitspiration literature (Tiggemann and Zaccardo, 2015) must be considered. Whereas Tiggemann and Zaccardo (2015) used generic fitspiration images, the present study used images of actual same-sex Korean SOCIAL MEDIA influencers. Prior exposure to culturally familiar influencers may have modulated the affective feedback system or generated identification effects, mitigating the threat response that unfamiliar idealized images elicit. Furthermore, unlike prior studies that focused exclusively on women, the present study included men, and the consistent direction of post-exposure negative affect reduction across both sexes suggests the possibility of a common mechanism transcending gender.

Third, the positive associative effect of health-oriented content may be relevant. As groups exposed to body-diversity content have shown more positive responses (Nie, 2026; Ogden et al., 2020; Stein et al., 2024), health-oriented influencer images may be processed as providing inspiration for health behaviors rather than mere appearance comparison. The absence of significant change in positive affect (PANAS-P) suggests that the decrease in negative affect may reflect a weakening of threat responses rather than the activation of positive affect.

Nonetheless, it is important to emphasize that this short-term reduction in negative affect does not imply that health-oriented influencer content is harmless overall. Health influencers may reduce negative affect in the short term while gradually promoting the internalization of highly normativized body ideals over the long term—a double-edged quality. The single-exposure design of the present study is insufficient to capture such cumulative effects, and longitudinal research involving repeated exposures is warranted.

### 4.3 Relationships between psychological variables and gaze metrics: gender differences

A key finding of this study is that the associations between psychological variables and gaze patterns toward specific body parts differed markedly by gender. In males, subjective BIP and BIP discrepancy significantly predicted chest fixation duration; in females, negative affect (PANAS2: upset) significantly predicted fixation on the chest and trunk back. These patterns are consistent with the theoretical predictions of drive for muscularity (McCreary and Sasse, 2000) and objectification/hypervigilance frameworks (Fredrickson and Roberts, 1997; McKinley and Hyde, 1996), respectively. However, because body surveillance and muscularity drive were not directly measured in the present study, these interpretations are offered as theoretically informed, exploratory inferences rather than as direct tests of these constructs. The findings suggest that cognitive factors may contribute more prominently to gaze allocation in males, whereas affective factors may play a comparatively larger role in females — a hypothesis that warrants further investigation using validated instruments such as the Objectified Body Consciousness Scale (OBC; McKinley and Hyde, 1996) and the Drive for Muscularity Scale (DMS; McCreary and Sasse, 2000).

#### 4.3.1 Male group: associations with body image perception

The finding that BIP discrepancy negatively predicted chest fixation duration in males indicates that the greater the extent to which a male perceives his current body as falling short of his ideal (i.e., the stronger his desire for a more muscular physique), the more selectively he attends to the influencer’s chest part. This finding is closely linked to the drive for muscularity concept (McCreary and Sasse, 2000). The core of drive for muscularity is preoccupation with the discrepancy between one’s current body and an ideal muscular physique; the larger this discrepancy, the greater the motivation to process the influencer’s muscle development parts—particularly the chest as a key indicator of muscle mass—as an information source for comparison with one’s body goals. From a goal-stimulus congruence perspective, when the goal of becoming more muscular is activated, the influencer’s chest part functions as a stimulus carrying information directly relevant to that goal, monopolizing attentional resources. This is consistent with the prediction from social comparison theory that stronger upward comparison motivation leads to increased attention to the comparison-relevant attributes of the target (Couture Bue, 2020; Festinger, 1954). The hierarchical regression finding that subjective BIP and BIP discrepancy together predicted chest fixation duration demonstrates that both subjective body size perception and the objective gap between current and ideal body shape independently influence gaze allocation. Thus, males’ processing of influencer body images can be understood as a cognitively driven body evaluation process.

Separately, the significant positive correlation between height satisfaction change (ΔBS8) and shin AOI fixation frequency in males is also noteworthy. This indicates a tendency for males who fixated more frequently on the influencer’s shin part to show increased height satisfaction after exposure. This pattern suggests that males may have experienced assimilative upward comparison, using the influencer’s body proportions (shin length in relation to overall height) as a positive reference and perceiving similar proportions as personally achievable—an experience that generated positive motivation and resulted in increased height satisfaction. This result illustrates that males’ processing of SOCIAL MEDIA influencer images is not a uniform threat response, but rather a complex process in which threat and inspiration operate simultaneously depending on the part being compared and the individual’s goals.

#### 4.3.2 Female group: associations with affective factors

The finding that negative affect (PANAS2: upset) significantly and positively predicted fixation duration on the influencer’s chest and trunk back parts in females reflects an emotion-driven attentional bias mechanism distinct from that observed in males. This result is theoretically linked to objectification theory (Fredrickson and Roberts, 1997) and the concept of body surveillance (McKinley and Hyde, 1996). Women with high body surveillance may experience automatic orientation of attention toward external stimuli associated with body shape anxiety (the influencer’s upper body) under negative affective states.

According to the hypervigilance model discussed in attentional bias research, individuals with higher negative affect or anxiety show automatic amplification of attention toward threatening or personally relevant stimuli. The finding that women with higher PANAS2 scores fixated longer on the influencer’s chest and trunk back supports an approach-type attentional bias (hypervigilance) rather than avoidance. This contrasts somewhat with findings by Scott et al. (2023) reporting avoidance of body-anxiety-related parts in women with body dissatisfaction, suggesting that the direction of attentional bias—hypervigilance (approach) vs. avoidance—may vary as a function of affective state and individual characteristics. Specifically, the influencer’s upper body part may simultaneously function as a threatening appearance comparison stimulus for women while paradoxically eliciting repeated attentional capture—a hypervigilance response—under emotionally uncomfortable states. This pattern suggests that women experiencing negative affect may be adopting an emotion-driven body surveillance strategy, using the influencer’s upper body to indirectly evaluate and compare their own physique.

The finding in females that upper body satisfaction (BS6) positively predicted thigh AOI fixation frequency indicates that women who are satisfied with their upper body tend to fixate more frequently on the influencer’s thigh part. This may reflect that women satisfied with a relatively non-anxiety-provoking body part (upper body) have the psychological resource availability to freely attend to lower body ideals as well. That is, psychological resource availability associated with one body part may enable exploratory attentional freedom toward other parts.

Taken together, the results confirm a gender-specific psychological mechanism in which males engage in a cognitively driven comparison process based on body image perception, while females show body surveillance-driven attentional bias modulated by affective state—with these distinct pathways expressed through gaze allocation patterns toward influencer body images. This demonstrates that the associations between psychological variables and visual attention can differ qualitatively by gender, underscoring the need for gender-specific approaches in media literacy education and clinical interventions.

### 4.4 Strengths

This study has several notable strengths. First, the use of actual Korean Instagram influencer photographs as visual stimuli enhanced ecological and cultural validity relative to studies using constructed or non-culturally specific images, although the laboratory-based procedure does not fully replicate naturalistic social media browsing. Second, the inclusion of both male and female participants allowed gender-comparative analysis — an important contribution given that most eye-tracking body image research has focused exclusively on women. Third, the systematic integration of eye-tracking metrics with psychometric data (BIP, BS, PANAS) provided a multimethod approach that limits reliance on self-report alone. Fourth, the application of FDR correction and VIF diagnostics improved the statistical rigor of the correlation and regression findings. Fifth, the counterintuitive finding of post-exposure decreases in negative affect contributes empirically to the debate about whether health-oriented influencer content functions similarly to conventional thin-ideal imagery.

### 4.5 Limitations and future directions

Several limitations of this study warrant careful consideration. First, and most importantly, key theoretical constructs referenced in the discussion — body surveillance, body shame (McKinley and Hyde, 1996), muscularity drive (McCreary and Sasse, 2000), and appearance internalization (Schaefer et al., 2015) — were not directly measured. Objectification theory and social comparison theory were employed as interpretive frameworks, not as operationalized constructs. Consequently, the findings should be understood as exploratory and theoretically informed rather than as direct tests of these theories. Future studies should incorporate the Objectified Body Consciousness Scale (OBC), the Drive for Muscularity Scale (DMS), and the SATAQ-4 alongside eye-tracking methods to enable more direct theory testing.

Second, social media use frequency and usage patterns (e.g., active/passive use, appearance-related activities, time spent on Instagram) were not assessed. Given that Instagram use frequency has been shown to predict visual attention to high-anxiety body regions (Couture Bue, 2020) and is consistently associated with body image concerns (Holland and Tiggemann, 2016), the absence of this variable limits the interpretability of individual differences in gaze patterns. Future research should include social media use as a covariate or moderator.

Third, the absence of a formal *a priori* power analysis means that some exploratory analyses, particularly those involving multiple AOI comparisons, may have had insufficient statistical power. The *post hoc* power estimate of approximately 1 − *β* = 0.72 for the Gender × AOI interaction falls below the recommended threshold of 0.80.

Fourth, the sample consisted of young Korean adults with normal-range BMI recruited via convenience sampling, limiting generalizability to individuals with weight-related concerns, older adults, and non-Korean cultural contexts. Fifth, the single-session, 300-s exposure design precludes examination of cumulative or long-term effects of influencer body image exposure. Sixth, the brief 10-item PANAS form may have measured affective states more restrictively than the full 20-item version. Seventh, influencer familiarity and habitual social media use were not controlled; thus, prior exposure effects cannot be excluded. Finally, the use of exclusively male research assistants during data collection, while intended to standardize the environment, may have introduced social evaluation concerns for female participants that cannot be fully ruled out.

Future research should address these gaps by incorporating validated measures of body surveillance, muscularity drive, and social media use; using larger, more diverse samples; and adopting repeated-exposure or longitudinal designs to examine cumulative effects of influencer body image consumption.

## 5 Conclusion

This exploratory eye-tracking study demonstrated that visual attention patterns toward Korean Instagram sports and health influencer body images are associated with psychological variables in adults with normal-range BMI, and that these associations differ by gender. In males, body image perception variables (BIP discrepancy and subjective BIP) were associated with gaze allocation toward the influencer’s chest, a pattern consistent with theoretical predictions related to muscularity drive. In females, pre-exposure negative affect predicted fixation duration on the influencer’s upper body, a pattern consistent with affective attentional bias frameworks. Furthermore, negative affect decreased significantly following influencer photo exposure, while body image perception and body satisfaction were not immediately altered.

Because body surveillance, muscularity drive, and social comparison tendency were not directly measured, the present findings should be interpreted as theoretically informed and exploratory rather than as confirmatory tests of objectification, drive for muscularity, or social comparison theories. These findings nonetheless indicate that the psychological impact of social media influencer content is not uniform, and that the direction and magnitude of visual attention may vary as a function of individual psychological characteristics and gender. This underscores the importance of gender-specific approaches in media literacy education, clinical interventions, and social media content policy. Future research should incorporate direct measurement of theoretically relevant constructs, diverse samples, and longitudinal designs to confirm and extend these preliminary findings.

## Data Availability

The raw data supporting the conclusions of this article will be made available by the authors, without undue reservation.

## Supplementary material

SUPPLEMENTARY FIGURE S1 Schematic illustrations of female influencer stimuli used in the experiment. Fifteen schematic line drawings, derived from photographs of Korean female sports and health Instagram influencers, are arranged in a 3 × 5 grid. The original photographs were selected based on predefined criteria, including follower count (≥10,000), sports/fitness content focus, and standardized pose. Schematic illustrations were created to preserve body posture and shape information while omitting photographic and facial detail for copyright and privacy protection. Each corresponding image was presented to participants for 20 seconds in a randomized order during the eye-tracking session.

SUPPLEMENTARY FIGURE S2 Schematic illustrations of male influencer stimuli used in the experiment. Fifteen schematic line drawings, derived from photographs of Korean male sports and health Instagram influencers, are arranged in a 3 × 5 grid. Selection criteria and presentation parameters were identical to those applied to female stimuli (see Supplementary Figure S1).
