# Supplementary figures and images for: Gender differences in gaze patterns to Instagram influencer body parts: predicted by subjective body image perception and negative affect
Source: Front Psychol. 2026 Jul 2;17:1857912. doi: 10.3389/fpsyg.2026.1857912 (PMC13382810; doi:10.3389/fpsyg.2026.1857912)

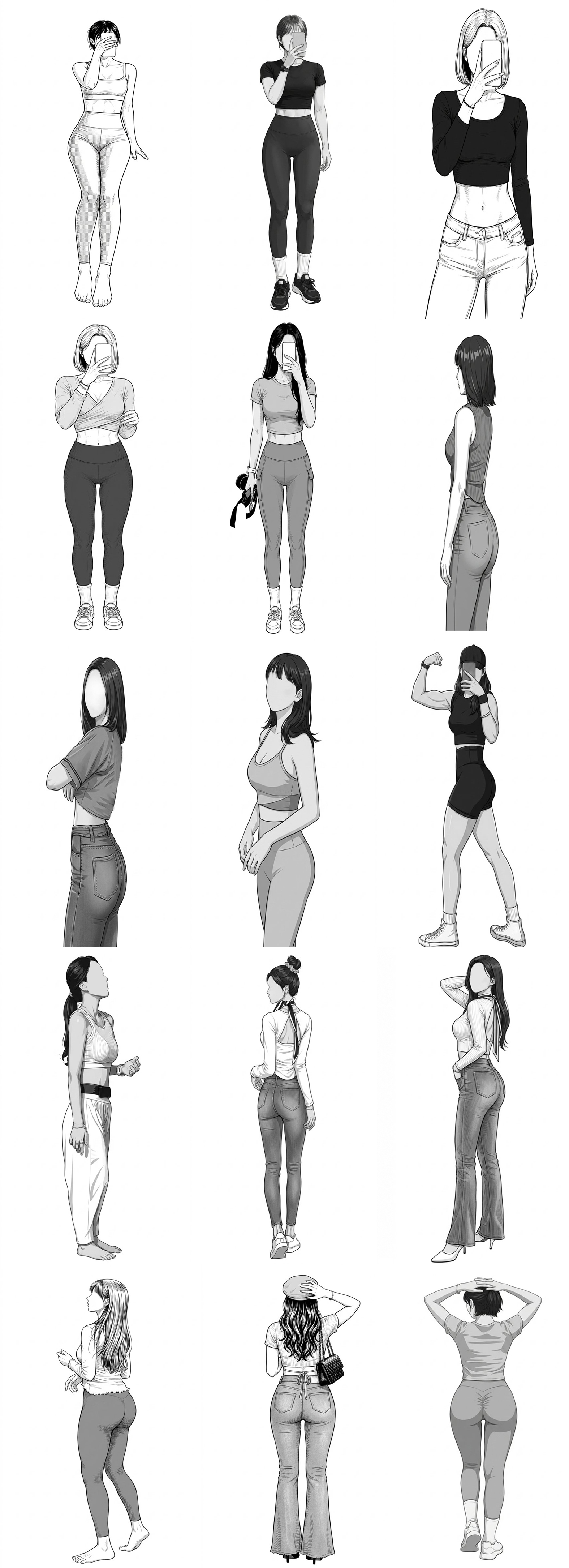

Supplement: SUPPLEMENTARY FIGURE S1 — Schematic illustrations of female influencer stimuli used in the experiment. Fifteen schematic line drawings, derived from photographs of Korean female sports and health Instagram influencers, are arranged in a 3 × 5 grid. The original photographs were selected based on predefined criteria, including follower count (≥10,000), sports/fitness content focus, and standardized pose. Schematic illustrations were created to preserve body posture and shape information while omitting photographic and facial detail for copyright and privacy protection. Each corresponding image was presented to participants for 20 seconds in a randomized order during the eye-tracking session. [file Image_1.jpg]

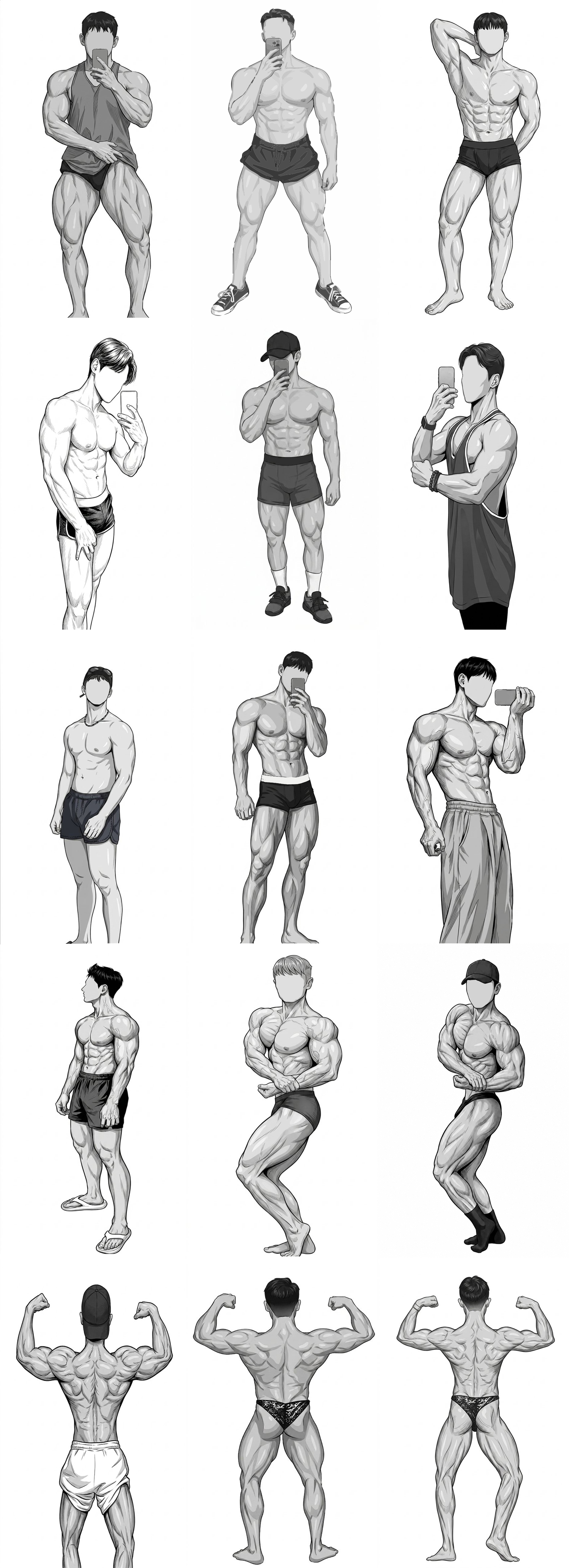

Supplement: SUPPLEMENTARY FIGURE S2 — Schematic illustrations of male influencer stimuli used in the experiment. Fifteen schematic line drawings, derived from photographs of Korean male sports and health Instagram influencers, are arranged in a 3 × 5 grid. Selection criteria and presentation parameters were identical to those applied to female stimuli (see Supplementary Figure S1). [file Image_2.jpg]
